# Linking morphology, genome, and metabolic activity of uncultured magnetotactic Nitrospirota at the single-cell level

**DOI:** 10.1186/s40168-024-01837-6

**Published:** 2024-08-24

**Authors:** Runjia Ji, Juan Wan, Jia Liu, Jinbo Zheng, Tian Xiao, Yongxin Pan, Wei Lin

**Affiliations:** 1 Key Laboratory of Earth and Planetary Physics, Institute of Geology and Geophysics, Chinese Academy of Sciences, Beijing, 100029 China; 2 France-China Joint Laboratory for Evolution and Development of Magnetotactic Multicellular Organisms, Chinese Academy of Sciences, Beijing, 100029 China; 3 College of Earth and Planetary Sciences, University of Chinese Academy of Sciences, Beijing, 100049 China; 4 CAS Key Laboratory of Marine Ecology and Environmental Sciences, Institute of Oceanology, Chinese Academy of Sciences, Qingdao, 266071 China; 5 Engineering Laboratory for Deep Resources Equipment and Technology, Institute of Geology and Geophysics, Chinese Academy of Sciences, Beijing, 100029 China

**Keywords:** Nitrospirota, Magnetotactic bacteria, Target-specific mini-metagenomics, NanoSIMS, Ecophysiology

## Abstract

**Background:**

Magnetotactic bacteria (MTB) are a unique group of microorganisms that sense and navigate through the geomagnetic field by biomineralizing magnetic nanoparticles. MTB from the phylum Nitrospirota (previously known as Nitrospirae) thrive in diverse aquatic ecosystems. They are of great interest due to their production of hundreds of magnetite (Fe_3_O_4_) magnetosome nanoparticles per cell, which far exceeds that of other MTB. The morphological, phylogenetic, and genomic diversity of Nitrospirota MTB have been extensively studied. However, the metabolism and ecophysiology of Nitrospirota MTB are largely unknown due to the lack of cultivation techniques.

**Methods:**

Here, we established a method to link the morphological, genomic, and metabolic investigations of an uncultured Nitrospirota MTB population (named LHC-1) at the single-cell level using nanoscale secondary-ion mass spectrometry (NanoSIMS) in combination with rRNA-based *in situ* hybridization and target-specific mini-metagenomics.

**Results:**

We magnetically separated LHC-1 from a freshwater lake and reconstructed the draft genome of LHC-1 using genome-resolved mini-metagenomics. We found that 10 LHC-1 cells were sufficient as a template to obtain a high-quality draft genome. Genomic analysis revealed that LHC-1 has the potential for CO_2_ fixation and NO_3_^−^ reduction, which was further characterized at the single-cell level by combining stable-isotope incubations and NanoSIMS analyses over time. Additionally, the NanoSIMS results revealed specific element distributions in LHC-1, and that the heterogeneity of CO_2_ and NO_3_^−^ metabolisms among different LHC-1 cells increased with incubation time.

**Conclusions:**

To our knowledge, this study provides the first metabolic measurements of individual Nitrospirota MTB cells to decipher their ecophysiological traits. The procedure constructed in this study provides a promising strategy to simultaneously investigate the morphology, genome, and ecophysiology of uncultured microbes in natural environments.

Video Abstract

**Supplementary Information:**

The online version contains supplementary material available at 10.1186/s40168-024-01837-6.

## Introduction

With the development of microscopy and sequencing techniques, the morphological and (meta)genomic identification and characterization of environmental microbes have been greatly advanced. However, verification of metabolic features that are predicted from the obtained genomic data, as well as the quantitative information on the ecophysiology of the individual uncultured microbes, remains very challenging. Nanoscale secondary-ion mass spectrometry (NanoSIMS) is a powerful tool that can be used to measure the distribution of stable isotopes in the microbes at a single-cell resolution. It allows the uptake of stable-isotope labeled substrates to be monitored over time and directly linking individual cells to their phylogenies and metabolic activities in the environment by combining with molecular identification tools, such as fluorescence *in situ* hybridization (FISH) [1–4].

Magnetotactic bacteria (MTB) are a group of microorganisms that sense and navigate along the geomagnetic field. This unique ability of MTB is endowed by the production of intracellular magnetic nanoparticles of magnetite (Fe_3_O_4_) and/or Greigite (Fe_3_S_4_), defined as magnetosomes [5, 6]. Magnetosome crystals are usually arranged in one or multiple linear chains within the cell, creating a permanent magnetic dipole moment and acting as a type of magnetoreceptor for the cell [7]. MTB are commonly found in a wide range of aquatic ecosystems and have been proposed to play important roles in the global biogeochemical cycling of Fe, C, N, S, P, etc. [8]. At least 16 different phyla of MTB have been identified thus far, although only a few have been cultivated in the laboratory [9]. Therefore, high-resolution information on the genetics, metabolism, and evolution of MTB populations remains mainly based on a few cultured MTB species, such as *Magnetospirillum magneticum* strain AMB-1 (AMB-1) and *M*. *gryphiswaldense* strain MSR-1 (MSR-1), which have been studied in detail using molecular biology approaches. The verified metabolic activities and ecophysiological roles of MTB within other MTB phyla are very limited.

One of the most intriguing MTB phyla is the Nitrospirota phylum (previously known as Nitrospirae). In contrast to other MTB, they can produce many hundreds of (up to 1000) Fe_3_O_4_-type magnetosomes per cell, and the magnetosomes are normally arranged in multiple bundles of chains [10, 11]. Nitrospirota MTB were originally considered to live in restricted environments with limited cell abundance. However, recent studies have shown that they are actually quite abundant in various aquatic ecosystems, including freshwater [12–15], estuaries [8], marine [16, 17], hot springs [18–20], and acidic peatlands [21]. Therefore, Nitrospirota MTB could make important contributions to aquatic biogeochemical cycles and to the natural remanent magnetism of sediments. Cultivation of Nitrospirota MTB under controlled laboratory conditions has not yet been achieved, which could be due to a lack of critical information on their ecology, physiology, and key natural products for their growth. Previously, most diversity and ecology studies of Nitrospirota MTB have been based on 16S rRNA gene-based analyses; while more recently, omics-based studies provide an opportunity for predicting their metabolic potential [11, 16, 22–26]. Although the morphological and genomic investigations of Nitrospirota MTB have been greatly improved, to our knowledge, no literature has documented their verified metabolic activities and ecophysiological roles.

**Fig. 1:**
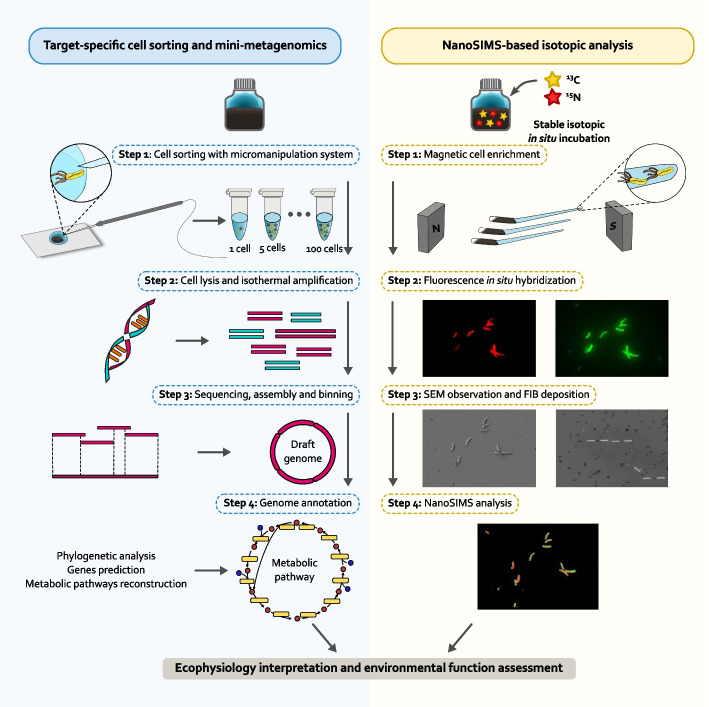
The pipeline developed in this study. For the target-specific cell sorting and mini-metagenomics, different numbers of magnetotactic bacteria (MTB) cells were sorted with a micromanipulation system (Step 1), which were then lysed and used as the template of whole genome amplification (Step 2). After sequencing, assembly, and binning, the draft genome of the MTB population was obtained (Step 3). Genome annotation and subsequent analysis (including phylogeny analysis, metabolism analysis) were then performed (Step 4). For the NanoSIMS-based isotopic analysis, the stable-isotope incubated MTB cells were first magnetically enriched (Step 1), then characterized by fluorescence *in situ* hybridization (FISH) (Step 2) and focused ion beam scanning electron microscope (FIB-SEM) (Step 3), and finally analyzed by NanoSIMS at the single-cell level (Step 4)

In this study, we developed a correlative pipeline that combines electron microscopy, FISH, target-specific mini-metagenomics, and NanoSIMS-based stable-isotope analysis to characterize the morphology, phylogeny, genome, and metabolic activity of uncultured MTB at the single-cell level (Fig. 1). We applied this pipeline to characterize an uncultured Nitrospirota MTB population (named LHC-1). We recovered the high-quality draft genome of LHC-1 and revealed that LHC-1 had the potential to fix carbon dioxide (CO_2_) and take up nitrate (NO_3_^−^) as a nitrogen source to produce energy and biomass. Furthermore, we uncovered the distribution of C, N, O, and S elements in LHC-1 cells over time and observed cell-to-cell heterogeneity of carbon and nitrogen uptake within LHC-1 population. Moreover, the carbon and nitrogen uptake rates appeared to be related to the growth status of LHC-1 cells. Overall, our study provides the first experimental evidence of carbon and nitrogen uptake by Nitrospirota MTB at the single-cell level, shedding new light on their metabolism and ecology in the natural environment.

## Materials and methods

### Sample collection and morphological observation

Sediment samples were collected from freshwater Lianhuachi Lake, Beijing, China (39°53′27″N, 116°18′52″E). The ambient air temperature during the sampling was about 25–30 °C. Briefly, surface sediments (5–10 cm) of the lake were collected using 600-ml plastic flasks and stored in the laboratory at room temperature. The sediment had a pH of 7.1 and a salinity of 0.52 ppt. A drop of sediment was observed using the “hanging-drop” method [27] under an Olympus BX51 microscope (Olympus Corporation, Japan) to check for the presence of MTB cells. For those samples containing MTB cells, the MTB cells were enriched using the “capillary racetrack” method [28].

For transmission electron microscopy (TEM) observation, 2 µL of enriched MTB cells was applied on a copper grid coated with formvar and carbon films (Beijing XXBR Technology Co., Ltd., China). After the grids were dried in a 35 °C incubator, they were washed with milliQ water and dried again in the 35 °C incubator. Then the dried cells were imaged on a JEM-2100 HR Electron Microscope (JEOL Ltd., Japan) at an accelerating voltage of 200 kV. The magnetosome crystal size quantification was performed as described previously [29]. Briefly, individual crystals were manually measured using the Fiji software [30]. The longest axis of the crystal is reported as crystal size or length, and the axis perpendicular to that is considered the width.

### PCR, cloning, and DNA sequencing

16S rRNA genes were PCR amplified from the magnetically enriched MTB samples using the bacterial universal primers 27F (5′-AGAGTTTGATCCTGGCTCAG-3′) and 1492R (5′-GGTTACCTTGTTACGACTT-3′) [31]. About 1400-bp PCR products were purified with the E.Z.N.A. Gel Extraction Kit (Omega Bio-Tek, USA) and ligated with the pMD19-T vector (TaKaRa, Japan), followed by transformation into DH5α competent cells and culturing on the LB agar plates containing ampicillin (100 ng/μL). Vector inserts from 10 randomly selected colonies were chosen for Sanger sequencing. The obtained sequences were clustered into operational taxonomic units (OTUs) at a threshold of 97% sequence identity using Mothur v1.43.0 [32].

### Single-cell sorting and whole genome amplification

A micromanipulation system was employed to separate MTB cells directly from the sediment. This system contains three parts: an inverted phase contrast microscope Olympus IX51 (Olympus Corporation, Japan), an IM-21 Microinjector (Narishige, Japan), and a MMO-202^ND^ Three-axis Joystick Type Oil Hydraulic Fine Micromanipulator (Narishige, Japan) (Fig. S1). The whole sorting process was conducted on a clean bench, including three main steps: (i) cell separation from sediment, (ii) cell washing, and (iii) cell counting and cryopreservation. Firstly, we used a sterilized glass slide as a sorting stage, and ferrite magnets were placed on the left side of the slide to create a magnetic field with the south pole of the magnet close to the sample (Fig. S1). A drop of sediment was placed onto the right end of the slide, and a drop of filter-sterilized sample water was then placed next to it. Using a Pasteur pipette, two drops were linked together. Under the magnetic field, MTB cells swam to the edge of the water droplet and were then collected using the micromanipulator. For the washing step, enriched cells were released into a filter-sterilized sample water and recollected at the edge of the droplets. Then, this washing step was repeated one time with filter-sterilized sample water and two more times with sterilized 1X PBS solution. Finally, six 200-μl centrifuge tubes containing 2 μl of 1X PBS solution with 1, 5, 10, 20, 50, and 100 LHC-1 cells, respectively, were cryopreserved at −20 °C until further processing.

For each centrifuge tube, whole genome amplification was conducted using the multiple displacement amplification (MDA) method with φ29 polymerase (REPLI-g Single Cell Kit, Qiagen). Cells were first lysed at 65 °C for 10 min using the lysis buffer, and a master mix containing φ29 polymerase was added into the tube (the final reaction volume was 50 μL). The amplification was performed for 8 h at 30 °C and heated to 65 °C for 10 min to stop the reaction. Amplified DNA was extracted using the Mag-MK PCR Products Purification Kit (Sangon Biotech, China), and the length of DNA fragments was examined by gel electrophoresis.

### Genomic sequencing, assembly, and annotation

Genomic sequencing, assembly, and annotation were performed as described previously [24]. Briefly, shotgun sequencing of six samples was performed using Illumina HiSeq 2500 using the pair-end 125 × 125 library with a 600-bp insert size (Beijing Genomics Institute, China). For each sample, the adapter sequences and low-quality bases were removed from the raw reads, and the cleaned reads were assembled using metaSPAdes v3.13.0 [33] with the following parameters: --only-assembler -k 31, 41, 51, 61, 71, 81, 91, 101, 111, and 121. Coverage information was determined using Bowtie2 v2.3.4.3 [34] and SAMtools v1.6 [35]. Thereafter, the assembled contigs and scaffolds longer than 2500 bp were binned using MetaBAT v0.26.1 [36] (--verysensitive mode). The quality of the draft genomes was assessed by CheckM v1.0.12 [37] using the “lineage_wf” workflow, CheckM2 v1.0.1 [38] with the “specific” neural network model, and BUSCO v5.6.1 [39] with bacteria_odb10 (2024-01-08) lineage dataset using default parameters. Finally, the draft genomes were annotated using Prokka v1.11 [40] with default parameters. The candidate magnetosome gene clusters (MGCs) were first identified using MagCluster v0.2.2 [41] with minimum number of magnetosome genes in a given contig set to 3 (“--threshold 3”) and then manually checked using the BLASTp program against the NCBI nonredundant protein sequences (nr) database [42]. The general genomic features were evaluated by QUAST v5.0.2 [43]. 16S rRNA gene sequence was retrieved from the draft genome and compared with known MTB 16S rRNA gene sequences. The metabolic potential of the LHC-1 genome was analyzed using METABOLIC v4.0 [44] and KEGG [45] with the BlastKOALA [46] tool.

### Phylogeny and taxonomic classification analysis

The 16S rRNA genes of the two OTUs (LHC-1 and LHC-2) obtained from magnetically enriched cells were first searched against the NCBI’s nucleotide collection (nr/nt) database using BLASTn search (*E*-value < 1e-05) [47]. Then, closely related and representative MTB 16S rRNA genes that were previously published were downloaded, and a total of 34 16S rRNA gene sequences were used to construct the phylogenetic tree. Briefly, sequences were first imported into MEGA v11.0.13 [48] and aligned with ClustalW [49]. The aligned sequences were trimmed to remove the poorly aligned regions, and the tree was then built using the MEGA phylogeny module under the maximum composite likelihood model with the bootstrap value set to 1000. The tree was rooted with *Thermodesulfobacterium geofontis* OPF15 (GenBank accession numbers CP002829.1) and *Thermodesulfobacterium commune* DSM 2178 and (CP008796.1), visualized using Interactive Tree Of Life (iTOL v6) [50], and finalized using the open-source vector graphics editor Inkscape (https://inkscape.org/).

To further infer the phylogenetic placement of LHC-1 in the Nitrospirota phylum, we performed phylogenomic and taxonomic classification analysis. For phylogenomic analysis, all previously published Nitrospirota MTB genomes (*n* = 41) and 159 species-representative (according to GTDB database release 214) non-MTB genomes belonging to the Nitrospirota phylum were downloaded from GenBank database. The 120 single-copy marker proteins [51] in those genomes were identified using GTDB-Tk (v2.1.0) [52] “identify” method. Multiple sequence alignment file of concatenated 120 single-copy marker proteins was then generated using GTDB-Tk “align” method. Finally, the phylogenomic tree was built using IQ-TREE (v2.0.3) [53] with a “TEST” option for the best-fit substitution model selection (LG + F + I + G4) and ultrafast bootstrap value set to 1000. The tree was then visualized using iTOL (v6) [50]. To test LHC-1’s novelty, pairwise average nucleotide identity (ANI) analysis was performed using FastANI (v1.1) [54] where all previously published Nitrospirota MTB genomes, along with the LHC-1 genome, were used as both the query genomes and reference genomes. To examine the pairwise ANI analysis result, we developed a Python package pairwiseANIviz (v1.0) (https://github.com/RunJiaJi/pairwiseANIviz) which is specialized for pairwise ANI visualization. The ANI value matrix was then visualized using pairwiseANIviz (v1.0), and a heatmap of ANI values was generated. Both the phylogenomic tree and ANI heatmap were finalized using the open-source vector graphics editor Inkscape (https://inkscape.org/).

### Stable-isotope incubation

For NanoSIMS analysis of Nitrospirota MTB cells, the collected sediment samples were first pooled and gently mixed and then equally divided into 15 subsamples (~25 mL), which were further kept at room temperature for 5 days in the dark for the recovery of local microenvironments and the increase of Nitrospirota MTB cells. Each of the 15 subsamples contained about 17 mL of sediment and 8 mL of lake water. All subsamples were examined under a light microscope to ensure the existence of rod-shaped Nitrospirota MTB cells. Then, the isotopes incubation started by adding 112.5 μL of 1-M Na^15^NO_3_ (98 atom% ^15^N, Sigma-Aldrich) and 50 μL of 1-M NaH^13^CO_3_ (98 atom% ^13^C, Sigma-Aldrich) into each subsample. MTB cells were magnetically enriched using the “capillary racetrack” method [28] at 1 h, 2.5 h, 8 h, 11 h, 16.5 h, 17.5 h, and 23 h, respectively. The enriched cells were washed twice with milliQ water and finally resuspended with 50-μL milliQ water.

### Fluorescence in situ hybridization (FISH)

After 1 h of isotope incubation, the MTB cells were enriched and fixed with 4% paraformaldehyde at 4 °C for 8 h. At the same time, untreated *Escherichia coli* (*E. coli*) strain DH5a cells were also fixed with 4% paraformaldehyde at 4 °C for 8 h and used as a negative control. Both types of fixed cells were pelleted and resuspended in 100 μL of 50% ethanol in 1X PBS and stored at −20 °C until further processing. Fixed MTB and *E. coli* cells were mixed and placed on a silicon wafer (one inch in diameter, Beijing Jingmei Hongye Technology Co., Ltd.), then air-dried at room temperature, and subsequently dehydrated using ethanol at different concentrations (50, 80, and 100%, with each concentration applied for 3 min). Two probes were used for hybridization. One was a specific probe labeled with Cy3 (an orange fluorescent dye). This probe, named BTC19, was designed based on the 16S rRNA gene sequence retrieved from the draft genome of LHC-1 cells (5′-*Cy3*-ACTATGATCCGTTCGACC-3′). Another one was the universal bacterial probe EUB338 labeled with a green fluorescent dye FAM (5′-*FAM*-GCTGCCTCCCGTAGGAGT-3′) [55]. For hybridization experiments, 9 μL of hybridization buffer was applied at each dried cell spot, and 1 μL of each probe was added to the hybridization buffer. After keeping the silicon wafer at 46 °C for 3 h in the dark, the excess probes were removed by immersing the silicon wafer into wash buffer at 48 °C for 15 min. After washing with milliQ water, the silicon wafer was air-dried at room temperature. The cells on the silicon wafer were finally observed and photographed with an epifluorescence microscope Olympus Optical BX51 equipped with a DP70 digital camera system (Olympus Corporation, Japan).

### FIB-SEM and NanoSIMS analyses

The silicon wafer with enriched MTB cells was placed in a scanning electron microscope (SEM) equipped with a focused ion beam (FIB) system (Zeiss Auriga Compact FIB-SEM, Germany). Platinum (Pt) rectangles (15 μm in length, 1.5 μm in width) were deposited using the Pt ion beam under FIB mode to be used as a marker for regions of interest (ROI). After SEM observation and Pt deposition via FIB, the silicon wafer was then transferred into NanoSIMS 50L (Cameca, Gennevilliers, France). By tracking Pt marks, ROIs were easily relocated in the secondary electron image mode of NanoSIMS 50L. Selected ROIs were scanned by a primary Cs^+^ ion beam with a beam current of 1pA and a beam diameter of ~100 nm. The raster resolution is 256 × 256 pixels with a dwelling time of 5 ms per pixel. The raster sizes range from 5 to 41 μm, and the corresponding cell numbers range from 1 to 14. Secondary ion imags of ^12^C^−^, ^13^C^−^, ^12^C^14^N^−^, ^12^C^15^N^−^, ^16^O^−^, and ^32^S^−^ were obtained in parallel. For each ion, six planes were consecutively captured.

NanoSIMS data were analyzed using the ImageJ plugin OpenMIMS developed by the National Resource for Imaging Mass Spectrometry at Harvard [56]. For each ion, six planes were first drift-corrected and compressed into the final NanoSIMS image, and then the LHC-1 cells were manually outlined as ROIs. The isotope ratios of ^13^C^−^/^12^C^−^ and ^15^N^−^/^14^N^−^ (inferred from the ^12^C^15^N^−^/^12^C^14^N^−^ ratio) for each LHC-1 cells were calculated. LHC-1 cells from control bottles without isotope additions served as controls and natural abundance levels. The cell size (length and width) was also measured at the same time.

### Statistical analyses

All datasets were analyzed for normality using the Shapiro-Wilk test and considered normal distribution if *p* > 0.05. Unpaired Student *t*-tests (normally distributed datasets) or Mann-Whitney *U*-test (non-normally distributed datasets) were performed to identify the difference of intracellular isotopic ratios among seven different time points. The differences were considered significant if *p* < 0.05.

Considering the nonuniformity and systematic errors inherent in the experimental data, we employed a statistical fitting approach based on the bootstrap sampling algorithm [57]. Initially, 70% of the data at each time point were randomly sampled, and their medians were calculated as the statistical value for that specific time point. Subsequently, a second-order polynomial was performed to fit these statistical values at each time point in order to obtain the corresponding fitting parameters. Finally, this entire process was repeated 10,000 times to determine the fitting parameter with the highest frequency of occurrence as our ultimate fitting result. The method effectively mitigates the bias in fitting results through extensive bootstrap sampling, thereby providing a more accurate representation of the underlying patterns within experimental data.

## Results

### Morphological identification and phylogenetic diversity of MTB

**Fig. 2:**
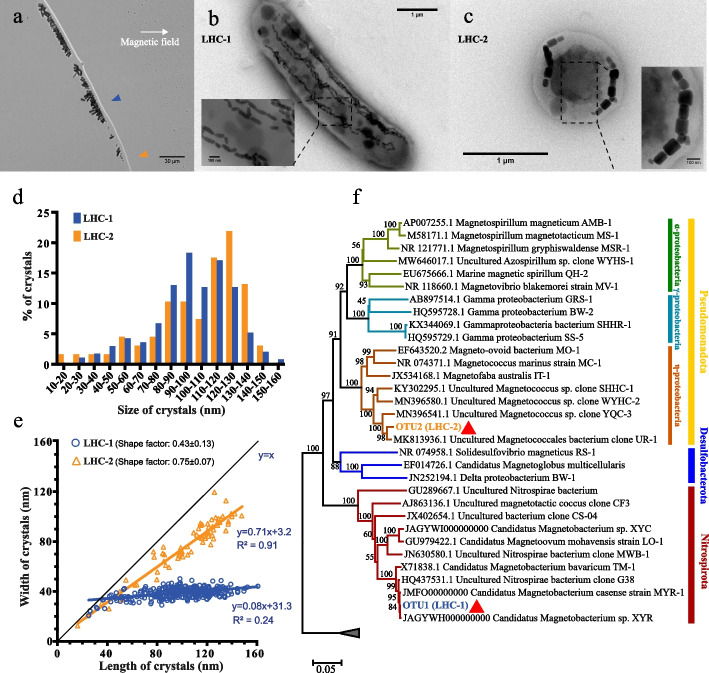
Morphological and 16S rRNA gene-based phylogenetic identification of two kinds of MTB. **a** Light microscopy image of MTB cells at the edge of a water droplet. The blue arrowhead indicates the slow-moving large rod-shaped MTB (LHC-1), and the orange arrowhead indicates the spherical-shaped fast-moving small magnetotactic cocci (LHC-2). **b** and **c** TEM images of a LHC-1 cell (**b**) and a LHC-2 cell (**c**). Insets of **b** and **c**: magnification of the magnetosomes in black dashed rectangles. **d** Crystal length distribution of LHC-1 and LHC-2 cells. **e** Shape factor (width/length ratio) of crystals in LHC-1 and LHC-2 cells. Each blue (LHC-1) and orange (LHC-2) dot represents one magnetosome. *n* = 320 (LHC-1) and 69 (LHC-2) in **d** and **e**, respectively. **f** Phylogenetic positions of OTU1 (LHC-1) and OTU2 (LHC-2) based on 16S rRNA gene sequences. Thirty-three previously published MTB 16S rRNA genes were used to construct the phylogenetic tree. The maximum likelihood phylogenetic tree was constructed using the MEGA v11.0.13 under the maximum composite likelihood model with the bootstrap value set to 1000. The bootstrap values of each node are indicated. Phyla names are illustrated on the right side of the figure. Besides, class names of phylum Pseudomonadota (previously known as Proteobacteria) were also illustrated, including *η-proteobacteria* (Magnetococcia according to the GTDB taxonomy), *α-proteobacteria*, and *γ-proteobacteria*. Branches belonging to different taxa are colored according to the names of different taxa

To search for MTB cells, the sediments of Lianhuachi Lake were collected and examined by a “hanging-drop” method [27]. MTB cells were then enriched by a “capillary racetrack” method [28] and examined by light and electron microscopy. We observed two different types of MTB cells (Fig. 2a). The dominant type was rod-shaped cells named *L*ian*h*ua*c*hi-1 (LHC-1), and another type was spherical cells named *L*ian*h*ua*c*hi-2 (LHC-2).

TEM observation showed that LHC-1 cells (~5.0 μm in length and ~1 μm in diameter) produce a few hundred bullet-shaped magnetic crystals that are organized into two to four bundles of chains (Fig. 2b), which are morphologically similar to previously identified Nitrospirota MTB strains such as ‘*Candidatus* Magnetobacterium cryptolimnobacter’ (XYR) [58] (5–7 μm in length and 1–2 μm in diameter), ‘*Ca*. Magnetobacterium casensis’ (Mcas) [22] (6–8 μm in length and 1–3 μm in diameter), and ‘*Ca*. Magnetobacterium bavaricum’ (Mbav) [10] (8–10 μm in length and 1.5–2 μm in diameter). LHC-2 cells (~1.2 μm in diameter) produce prismatic-shaped magnetic crystals, which are organized into two separated single chains on the opposite side of the cell (Fig. 2c). The size distribution of magnetosome crystals in LHC-1 and LHC-2 is shown in Fig. 2d. LHC-1 represents a bimodal crystal size distribution with peaks centered in the 90–100 nm and 110–120 nm size ranges. LHC-2 also shows a bimodal crystal size distribution, but its peaks are centered in the 80–100 nm and 120–130 nm size ranges. The average length of the crystals in LHC-1 and LHC-2 is 98.6 ± 25.4 nm and 104.6 ± 29.6 nm, respectively. The shape factor of crystals (width/length ratio) differs between LHC-1 (0.43) and LHC-2 (0.75) cells (Fig. 2e). A further scatterplot and regression analysis of crystal width versus crystal length shows that the crystals in LHC-2 grow along both the length and width axis, indicating a more isotropic growth pattern. In contrast, the crystals in LHC-1 grow predominantly along the length axis, suggesting a more anisotropic growth pattern (Fig. 2e).

To identify the taxonomy of these two types of MTB, we employed 16S rRNA gene analysis of the magnetically enriched MTB cells. The 16S rRNA gene-based phylogenetic tree shows that the dominant amplicon (OTU1) was closely related to XYR [58] (99.9% identity), Mcas [22] (99.7% identity), uncultured Nitrospirota bacterium clone G38 [59] (99.6% identity), and Mbav [10] (98.1% identity) (Fig. 2f), indicating that the dominant LHC-1 cells likely belong to the Nitrospirota phylum, which was further confirmed by phylogenomic analysis and FISH (see more details below). The other less abundant amplicon (OTU2) was closely related to a previously identified uncultured Magnetococcales bacterium clone UR-1 (GenBank accession number MK813936, 98.2% identity) belonging to the ‘*Candidatus* Etaproteobacteria’ class (*Magnetococcia* according to the GTDB taxonomy) (Fig. 2f), indicating that the less abundant LHC-2 cells may be affiliated with the Etaproteobacteria class of the Pseudomonadota phylum. Given the significant interest in Nitrospirota MTB and the higher abundance of LHC-1 cells in the collected samples, we focused on the LHC-1 cells in this study.

### Genomic characterization and metabolism prediction of ‘*Candidatus* Magnetobacterium’ sp. LHC-1

To obtain its genome, LHC-1 cells were isolated from the sediment through single-cell sorting using a micromanipulation system (Step 1 on the left side in Fig. 1). We examined the minimum number of cells needed to reconstruct the draft genome of LHC-1. Thus, 1, 5, 10, 20, 50, and 100 LHC-1 cells were obtained from the sediment, respectively. Each cell population underwent whole genome amplification, sequencing, assembly, and binning (Steps 2 and 3 on the left side in Fig. 1). The results indicate that high- to medium-quality draft genomes (completeness > 70% and contamination < 5%) could be reconstructed from samples containing 10 or more LHC-1 cells (Table 1). The pairwise average nucleotide identity (ANI) values of genomes reconstructed from 10 or more cells were above 99.99%, indicating that they originated from the same MTB species, which was named as ‘*Candidatus* Magnetobacterium’ sp. LHC-1.

**Table 1:** Genome statistics of draft genomes reconstructed from different numbered cells of ‘*Candidatus* Magnetobacterium’ sp. LHC-1

Parameter	Groups of LHC-1 cells with different numbers					
	1 cell	5 cells	10 cells^a^	20 cells	50 cells	100 cells
Genome size (Mbp)	1.08	0.21	4.08	3.55	3.85	3.84
No. of scaffolds	140	31	283	345	150	295
N50 (kb)	11.009	7.250	22.734	14.774	41.955	20.486
GC content (%)	30.71	30.81	48.69	49.18	48.89	49.00
CheckM completeness (%)	1.72	7.76	94.83	83.07	86.21	84.33
CheckM contamination (%)	0	0.86	0	0.86	0	0
CheckM2 completeness (%)	22.8	16.29	98.73	82.55	88.92	91.18
CheckM2 contamination (%)	4.97	0.15	0.24	0.6	0.32	0.44
BUSCO completeness (%)	0.8	0	90.3	77.4	87.1	87.1
BUSCO contamination (%)	0	0	0	0	0	0
No. of coding sequence (CDS)	1250	261	3764	3272	3526	3583
No. of tRNAs	1	4	21	18	19	21
The presence of the 23S, 16S, and 5S rRNA genes^b^	False	False	True	False	True	True

^a^This genome sequence was selected for in-depth phylogenomic, genomic, and metabolic analyses

^b^Genome that encodes all 23S, 16S, and 5S rRNA genes is marked as true; if any of these rRNA genes is missing, then marked as false

Here, we chose the draft genome generated from 10 LHC-1 cells for further analyses due to its highest completeness and low contamination estimated by CheckM, CheckM2, and BUSCO (Table 1). The genome of LHC-1 is about 4.08 Mbp with an average GC content of 48.69%. It contains 283 scaffolds (the largest scaffold is 119.447 kb, and N50 = 22.734 kb), 3764 putative genes, 21 tRNA genes, and 3 rRNA genes. The 16S rRNA gene sequence from the genome of LHC-1 represents 100% identity to the 16S rRNA gene sequence of OTU1, confirming that the obtained genome was from the rod-shaped LHC-1 cells.

**Fig. 3:**
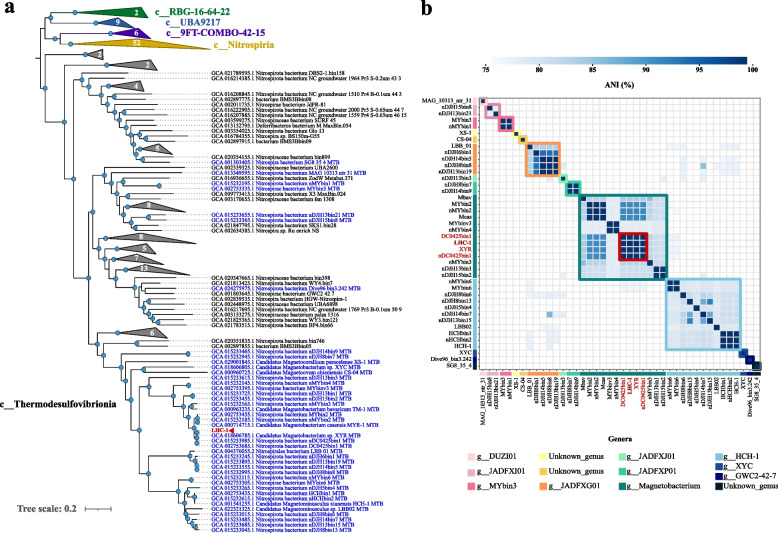
Genome-based taxonomic characterization of LHC-1. **a** Phylogenomic tree of Nitrospirota bacterial genomes including LHC-1, 41 MTB genomes, and 159 non-MTB genomes. The maximum-likelihood tree was constructed using IQ-TREE (v2.0.3) [53] under the LG+F+I+G4 substitution model based on the concatenated alignment of 120 single-copy marker proteins [51] generated with GTDB-Tk (v2.1.0) [52]. Nodes with bootstrap values larger than 75% are indicated with dots. Previously published MTB genomes were colored blue, while LHC-1 was colored red and indicated with a red triangle. The five classes (based on the GTDB taxonomy) under Nitrospirota were illustrated on the figure, and branches of each class are colored accordingly. **b** Heatmap of pairwise ANI values among all 42 Nitrospirota MTB genomes. Pairwise ANI was analyzed using FastANI (v1.1) [54] and visualized using pairwise ANI viz. (v1.0) (a Python package we developed in this study). Note that the classification analysis result from GTDB-Tk was integrated in the figure, and MTB genomes belonging to different genera were indicated using different colored bars and squares. LHC-1 and three previously published MTB genomes (DC0425bin1, XYR, and nDC0425bin1), which belong to the same species according to the ANI threshold of 95% [60], were highlighted in red

We performed a phylogenomic analysis of LHC-1 with 41 available Nitrospirota MTB genomes and 159 Nitrospirota non-MTB genomes based on 120 bacterial single-copy concatenated protein sequence alignments (Fig. 3a, see Supplementary file data sheet 1 for more information). Of the 120 single-copy marker genes examined, 110 were identified in the LHC-1 genome. For each of these markers in the LHC-1 genome, a blastp search was performed against the NCBI nr database. The results showed high similarities with the corresponding marker proteins of Nitrospirota microbes, which is consistent with the phylogenomic analysis using GTDB-Tk. Thus far, all reported draft genomes of Nitrospirota MTB belong to the Thermodesulfovibrionia class. The phylogenomic analysis result confirmed that LHC-1 is affiliated with the Nitrospirota phylum, and LHC-1 has a close relationship with the genomes of the morphologically identified XYR and Mcas strains, which is consistent with the result of 16S rRNA gene phylogenetic analysis (Fig. 2f). We further performed the ANI analysis of LHC-1 and available Nitrospirota MTB genomes (Fig. 3b, see Supplementary file data sheet 2 for more information), which indicates that LHC-1 and the other three previously published MTB genomes (XYR [58], DC0425bin1 [24], and nDC0425bin1 [21]) belong to the same species (> 95% ANI [60]).

**Fig. 4:**
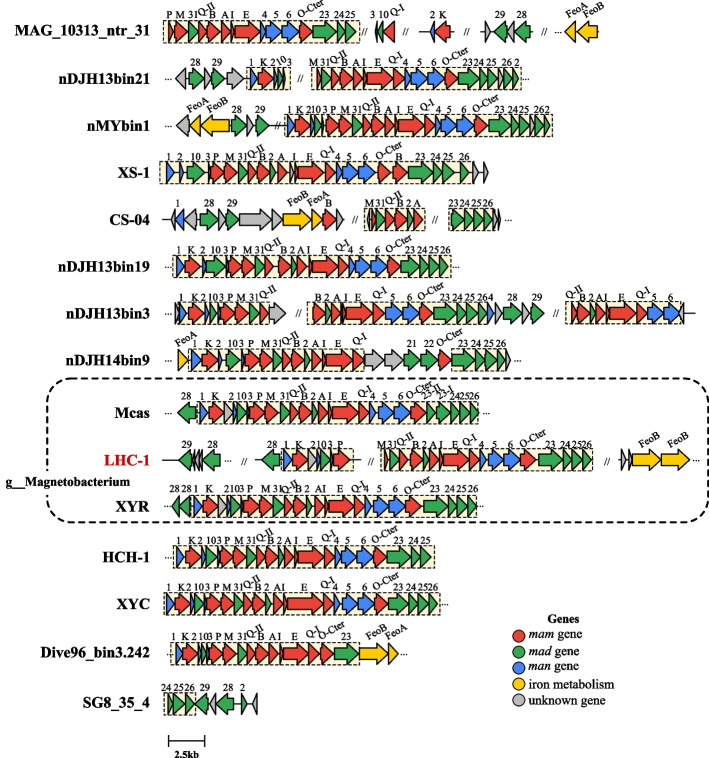
Schematic representation of MGCs in LHC-1 and previously reported representative Nitrospirota MTB. At least one representative MTB population from each genus within the Nitrospirota phylum was selected for comparison. Note that LHC-1 has a close phylogenetic relationship with populations of XYR and Mcas, which all belong to the same genus *Magnetobacterium*. The conserved magnetosome genes in Nitrospirota MTB are highlighted in light yellow boxes

Magnetosome gene cluster (MGC) involved in magnetosome biosynthesis and organization has been identified in the LHC-1 genome. The MGC region of LHC-1 contains 26 magnetosome genes, including 10 *m*agnetosome‐*a*ssociated *m*embrane (*mam*) genes of *mamK*, *-P*, *-M*, *-Q*^*(II)*^, *-B*, *-A*, *-I*, *-E*, *-Q*^*(I)*^, and *-O*^*-Cter*^, 10 *m*agnetosome‐*a*ssociated Deltaproteobacteria (*mad*) genes of *mad29*, *-28*^*(I)*^, *-28*^*(II)*^, *-10*, *-31*, *-2*, *-23*, *-24*, *-2*5, and *-26*, and 6 *m*agnetosome‐*a*ssociated Nitrospirota (*man*) genes of *man1*, *-2*, *-3*, *-4*, *-5*, and *-6* (Fig. 4, see Supplementary file data sheet 3 for more information). The gene content and order of magnetosome genes of LHC-1 are highly conserved with the other available Nitrospirota MGCs, and further NCBI BLASTp analysis revealed that all magnetosome genes from LHC-1 have significant hits with the magnetosome genes from the other known Nitrospirota MTB. The *man* genes (*man1-6*) have been proposed to contribute to the production of large numbers of magnetosomes per cell in Nitrospirota MTB [22]. We used several protein secondary structure prediction tools to analyze the Man1-6 proteins from LHC-1 (Table S1). The results show that Man1, -4, -5, and -6 proteins all contain coiled-coil domains. In MSR-1, a membrane protein CcfM (curvature-inducing coiled-coil filament interacting with the magnetoskeleton) was found to localize in a filamentous pattern along regions of the bacterial inner positive-cell curvature through its coiled-coil motifs and also to link the MamK magnetoskeleton to cell morphology regulation [61]. These data indicate a strong potential for the Man proteins of Nitrospirota MTB to be involved in complex magnetosome chain organization through their coiled-coil domains.

**Fig. 5:**
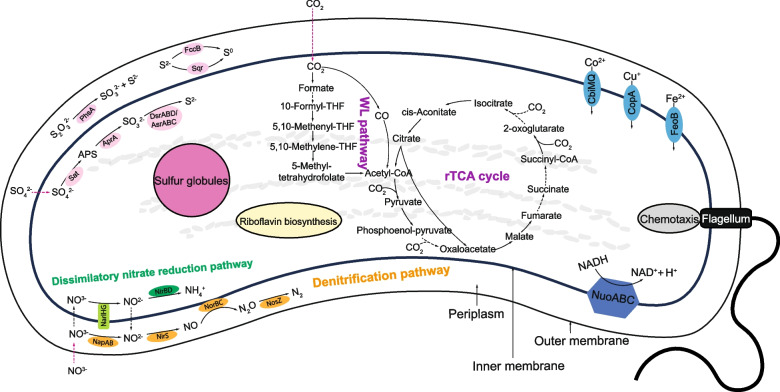
The metabolic cartoon constructed from the LHC-1 genome. The overall metabolic potential of LHC-1 was analyzed using METABOLIC (v4.0) [44], and the key enzymes that related to carbon, nitrogen, and sulfur metabolic pathways were double checked using KofamScan (v1.3.0) [71] against KOfam database. The draft genome of LHC-1 possesses a nearly complete Wood-Ljungdahl (WL) pathway, most enzymes for the reductive tricarboxylic acid (rTCA) cycle, and a complete denitrification pathway and dissimilatory nitrate reduction to ammonium pathway. The draft genome of LHC-1 also possesses a set of genes involving sulfur cycling, ion transportation, riboflavin biosynthesis, and chemotaxis. The black solid arrows indicate the presence of related enzyme genes in the draft genome of LHC-1. The black dashed arrows indicate the related enzyme genes are not discovered in the genome. The magenta dashed arrows indicate the predicted events that have not been confirmed or analyzed

The genome of LHC-1 contains genes encoding for almost all key enzymes associated with the Wood-Ljungdahl (WL) pathway (also called the reductive acetyl-CoA pathway) (Fig. 5). The WL pathway is the main mechanism for energy conservation and carbon fixation under anaerobic conditions in most archaea and some bacteria [62–68] and is thought to be one of the oldest CO_2_ fixation pathways to produce acetyl-CoA [69]. This finding is consistent with the previous identification of the WL pathway in the other Nitrospirota MTB [22, 23]. The LHC-1 genome also contains genes encoding most of the key enzymes of the reductive tricarboxylic acid (rTCA) cycle (Fig. 5). The rTCA cycle is basically a reverse version of tricarboxylic acid (TCA) cycle, and some bacteria use rTCA cycle to produce carbon compounds from CO_2_ and H_2_O [70]. Thus, these data suggest the metabolic potential of LHC-1 for autotrophic carbon fixation leading to the incorporation of CO_2_ into biomass.

For nitrogen metabolism, the draft genome of LHC-1 contains genes encoding two complete nitrate reduction pathways. One is the denitrification pathway, which includes the reduction of NO_3_^−^ via nitrite (NO_2_^−^), nitric oxide (NO), and nitrous oxide (N_2_O) to dinitrogen (N_2_) gas (NO_3_^−^→NO_2_^−^→NO→N_2_O→N_2_) (Fig. 5). In bacteria, this process is used as an alternative to oxygen respiration under low-oxygen or anoxic conditions [72, 73]. Another one is the dissimilatory nitrate reduction to ammonium pathway, which includes the reduction of NO_3_^−^ via NO_2_^−^ to NH_4_^+^ (Fig. 5). This pathway is also known as the NO_3_^−^ respiration process where NO_3_^−^ acts as an electron acceptor under anaerobic conditions [74, 75]. Together, these data indicate that LHC-1 might be able to use NO_3_^−^ as a nitrogen source and an electron acceptor to respirate and survive in hypoxic or anoxic environments.

Genomic prediction showed that LHC-1 cells have the genetic potential of sulfate reduction (SO_4_^2−^→APS→SO_3_^2−^→S^2−^), sulfide oxidation (S^2−^→S^0^), and thiosulfate disproportionation (S_2_O_3_^2−^→SO_3_^2−^+S^2−^) (Fig. 5). Consistently, we observed sulfur granules in the cytoplasm of LHC-1 cells (Fig. S2). Most LHC-1 cells showed no visible sulfur granules, while some contained a few or were completely filled with sulfur globules, representing a different sulfur metabolic status of LHC-1 cells.

### Correlative microbial identity, genome, and metabolism analyses of LHC-1

As the LHC-1 genomic analysis suggested the potential for CO_2_ fixation and NO_3_^−^ reduction, we incubated the LHC-1 cells with ^13^C-labeled bicarbonate and ^15^N-labeled nitrate to test its C and N metabolisms (Steps 1–4 on the right side in Fig. 1). To simultaneously correlate and characterize the identity, morphology, genome, and metabolism of LHC-1 at the single-cell level, we conducted a combination of FISH, FIB-SEM, and NanoSIMS analysis on the same cell using an electrically high-conductive single-crystal silicon wafer as a cell carrier.

**Fig. 6:**
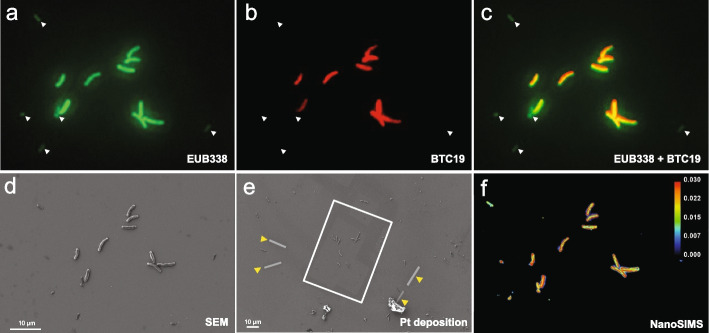
A combination of FISH, FIB-SEM, and NanoSIMS analyses. **a–c** LHC-1 cells were mixed with *E. coli* cells and incubated with NaH^13^CO_3_ for 1 h and then fixed and dried on a silicon wafer. Thereafter, the FISH experiment was conducted using a universal bacterial probe EUB338 (**a**) and an LHC-1-specific probe BTC19 (**b**), and the bacteria were imaged with an Olympus Optical BX51 fluorescence microscope. **c** A merged image of **a** and **b**. The white arrowheads point to *E. coli* cells in **a**–**c**. **d** SEM image of the same region of interest (ROI) of **a** to **c**, which was acquired at an accelerating voltage of 5 kV with a 6-mm working distance. The LHC-1 cells can be easily distinguished from *E. coli* due to their disparity in sizes, and the correspondence with the LHC-1-specific probe BTC19 in **b**. **e** Pt deposition (yellow arrowheads) on the ROI with FIB-SEM as markers for NanoSIMS imaging. The ROI analyzed using NanoSIMS is outlined as white rectangular. **f** NanoSIMS image of ^12^C^−^ of the same ROI

For the FISH experiment, LHC-1 cells and *E. coli* cells (as an internal control) were mixed and hybridized with two kinds of fluorescent probes (Fig. 6a–c). One probe is the universal bacterial probe EUB338 (false colored as green). Another probe is BTC19 (red), which targets specific regions of the 16S rRNA gene of LHC-1. As expected, both the large rod-shaped MTB cells and the *E. coli* cells (pointed by white arrowheads in Fig. 6a–c) were stained with the EUB338 probe. Only the rod-shaped MTB cells were specifically targeted by the BTC19 probe, confirming that the rod-shaped MTB cells are the sequenced LHC-1 cells.

To further pinpoint the LCH-1 cells and quantify the carbon and nitrogen metabolism, we performed FIB-SEM and stable-isotope incubation coupled NanoSIMS analysis (Fig. 6d–f) on the same sample cells in Fig. 6a–c. LHC-1 and *E. coli* cells can be easily distinguished in the SEM image (Fig. 6d) by correlating with the corresponding FISH images. To track the same cells for NanoSIMS analyses, the area near the ROI was marked with Pt deposition (Fig. 6e, pointed by yellow arrowheads) under the FIB mode of the FIB-SEM equipment. After Pt deposition, the ROI can be easily recognized during NanoSIMS analysis (Fig. 6f).

### NanoSIMS single-cell analysis

**Fig. 7:**
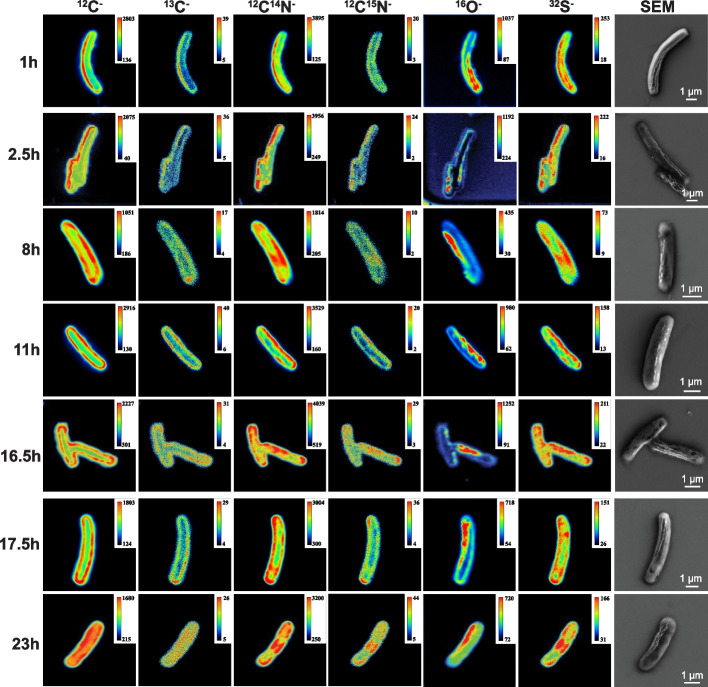
Representative NanoSIMS and SEM images at different time points after stable-isotope incubations. Isotope images were obtained with NanoSIMS. Six secondary ions (^12^C^−^, ^13^C^−^, ^12^C^14^N^−^, ^12^C^15^N^−^, ^16^O^−^, and ^32^S^−^) were detected at the same time under the Cs^+^ primary ion beam. For each isotope image, six serial secondary ion images (planes) were generated and averaged after drift correction. The calibration bar in each image indicates the secondary ion numbers collected in each pixel

To test for the uptake of CO_2_ and NO_3_^−^ from the environment into single LHC-1 cells, the NaH^13^CO_3_ and Na^15^NO_3_ were added to the collected sediment samples containing LHC-1 cells. The NanoSIMS analysis covered a total of 330 LHC-1 cells at 8 different incubation time points (0-, 1-, 2.5-, 8-, 11-, 16.5-, 17.5-, and 23-h post-incubation, hpi). At each time point, six isotopes (^12^C^−^, ^13^C^−^, ^12^C^14^N^−^, ^12^C^15^N^−^, ^16^O^−^, and ^32^S^−^) were detected simultaneously to generate six serial secondary-ion images (Fig. 7), which could reveal the physiological properties and metabolic activities of LHC-1 with a single-cell scale resolution.

Overall, all detected intracellular isotopes were found not uniformly distributed. The signals of the ^12^C^−^, ^13^C^−^, ^12^C^14^N^−^, ^12^C^15^N^−^, and ^32^S^−^ isotopes are predominantly distributed around the bacterial cells (Fig. 7). The ^13^C^−^ and ^15^N^−^ isotopes represent the fate of newly fixed carbon and nitrogen compounds. Apparently, the stable isotope incubated cells are substantially enriched in ^13^C^−^ and ^15^N^−^ isotopes, suggesting the active CO_2_ fixation and NO_3_^−^ reduction in LHC-1 cells. The magnetosome chains of LHC-1 cells are clearly visible in the SEM images, and they can be recognized under the ^16^O^−^ images because more ^16^O^−^ is retained in the Fe_3_O_4_ magnetosome chains than in the rest of the cell (Fig. 7). Interestingly, the ^32^S^−^ distribution is very similar to the ^12^C^14^N^−^ signals in the LHC-1 cells at all time points (Fig. 7), and we did not observe obvious sulfur granules in the SEM images of these cells.

**Fig. 8:**
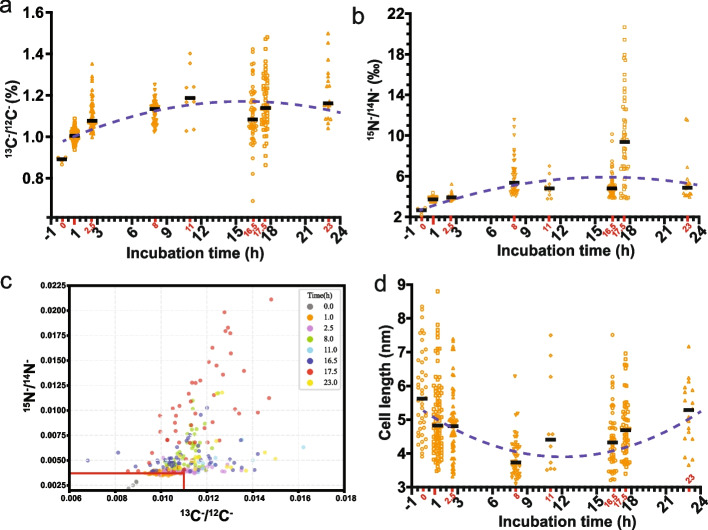
Carbon and nitrogen isotope ratios at different incubation time points. **a** and **b** Scatter dot plots (median in black lines) of the ^13^C and ^15^N atom percent enrichment within cells for each incubation time point, respectively. The dashed lines are the results of the fitting analyses with the residual sum of squares (RSS) of 0.0193 in **a** and 9.0457 in **b**. **c** Scatter plot of 330 LHC-1 cells’ carbon and nitrogen ratio in eight incubation time points. Each dot represents a single cell. Many of the data points are gathered around the lower left of the plot indicating their intracellular isotope levels are close to natural abundances. The natural environmental ^13^C^−^/^12^C^−^ ratio value 0.0112 and ^15^N^−^/^14^N^−^ ratio value 0.0037 are indicated as red lines. Both the plot and isotope images show that some cells of 8 h and 17.5 h tend to accumulate more ^15^N atoms, which may represent a unique metabolic status. **d** Scatter dot plots (median in black lines) of the cell length distribution for each incubation time point. The dashed line is the result of the fitting analyses with the RSS of 0.4715

We measured the ^13^C^−^/^12^C^−^ and ^15^N^−^/^14^N^−^ (derived from the ^12^C^15^N^−^/^12^C^14^N^−^ ratio) ratios of the controls and used them as a starting point to show the ^13^C^−^ and ^15^N^−^ enrichment in the LHC-1 cells after isotope incubation. Then, we plotted the ratio changes of ^13^C^−^/^12^C^−^ and ^15^N^−^/^14^N^−^ in LHC-1 cells at different incubation time points. The ^13^C^−^/^12^C^−^ ratio showed an increasing tendency during the early incubation period. Specifically, the ^13^C^−^/^12^C^−^ ratio gradually increased at 1, 2.5, 8, and 11 hpi, indicating active CO_2_ fixation at these time points (Fig. 8a). After 11 hpi, the regression fit line showed a plateau stage with a slight decrease between 11 and 16.5 hpi, followed by a slight increase between 16.5 and 23 hpi (Fig. 8a). Similarly, the ^15^N^−^/^14^N^−^ ratio also showed an increasing trend during the early incubation period (1, 2.5, and 8 hpi) (Fig. 8b), indicating the ability of LHC-1 to take up NO_3_^−^ as an N source for growth. After 8 hpi, the regression fit line showed a plateau phase with two decreases (8–11 hpi and 17.5–23 hpi) and one increase (16.5–17.5 hpi) (Fig. 8b). Together, these data indicate that LHC-1 is an autotrophic bacterium that produces its own food by CO_2_ fixation and generates energy by NO_3_^−^ assimilation.

A scatter plot of the ^13^C^−^/^12^C^−^ ratio versus the ^15^N^−^/^14^N^−^ ratio was plotted with a total of 330 LHC-1 cells (Fig. 8c). In the natural environment, the ^13^C^−^/^12^C^−^ ratio is approximately 0.0112, and the ^15^N^−^/^14^N^−^ ratio is approximately 0.0037 [76, 77] (red lines in Fig. 8c). At 0 hpi, the scatter is clustered in the lower left of the plot (in the red box). As time progressed, the scatter gradually spread out from the natural abundance range. In addition, the ^13^C^−^/^12^C^−^ and ^15^N^−^/^14^N^−^ ratios between individual LHC-1 cells at 0 and 1 hpi are concentrated, while the values at the other time points are relatively scattered and different. Moreover, this difference becomes more evident as the incubation time progresses, indicating obvious differences in metabolic status or different types of metabolism.

Finally, we found that the carbon and nitrogen uptake rates appeared to correlate with the growth status of LHC-1 cells. To uncover the relationships between C and N metabolism and cell length of LHC-1 cells, we used statistical fitting analysis with these data at the eight different time points (blue dashed lines on Fig. 8a, b, d). In the first 8 h, both C and N elements were gradually incorporated into the LHC-1 cells (Fig. 8a, b), while the length of LHC-1 cells was gradually decreased (Figs. 8d and S3 and Table S2), indicating the negative correlation of carbon and nitrogen uptake with cell length. At 0 hpi, the length of LHC-1 cells ranged from 3.9 to 8.4 μm, with the maximum length being almost twice the minimum length, indicating that LHC-1 are not synchronized in the natural environment but are at different growth stages. Previous studies have shown that the bacteria doubling time in the wild is much slower than that of laboratory-grown [78]. The average cell length value at 0 hpi (5.78 ± 1.24) is about 1.5 times higher than that at 8 hpi (3.88 ± 0.64), indicating the addition of extra inorganic C and N sources (H^13^CO_3_^−^ and ^15^NO_3_^−^) to the natural environment might trigger the accelerated growth of LHC-1, with a gradual decrease in cell length possibly due to the stimulation of cell division. Thus, the cell growth in the first 8 h correlates with the consistent C and N uptake during this time period, while the cell length gradually increased after 8 h (Fig. 8d), together with the slowing of the tendency of C and N uptake after 8 h (Fig. 8a and b).

## Discussion

Although cultivation-independent methods, such as 16S rRNA gene- and omics-based analyses, have provided general information on the diversity and metabolic potential of environmental Nitrospirota MTB, a deeper understanding of their metabolism and ecophysiology is still lacking. In this study, we developed a workflow to first obtain the high-quality genome of the uncultured Nitrospirota MTB LHC-1 by target-specific mini-metagenomics from a few cells and analyze the metabolic potential of LHC-1. Subsequently, we used NanoSIMS to test that LHC-1 could fix CO_2_ and use NO_3_^−^ as a nitrogen source. We also observed community dynamics and heterogeneity of C and N metabolism over time at the single-cell level. These studies have improved our understanding of the metabolism, ecophysiology, and biogeochemical dynamics of Nitrospirota MTB in the aquatic system.

### Combining target-specific mini-metagenomics and NanoSIMS

By combining magnetic selection and microscopy-based single cell sorting, we showed that 10 LHC-1 cells as template of whole genome amplification are sufficient to obtain a high-quality draft genome (Table 1). Indeed, single-cell sorting by micromanipulation followed by whole genome amplification through MDA has been previously used to obtain draft genomes of several other Nitrospirota MTB populations from the environments. For example, Jogler and colleagues have identified the MGC of ‘*Ca.* Magnetobacterium bavaricum’ (Mbav) using this approach together with PCR screening of metagenomic libraries [11]. Subsequently, Kolinko et al. [23] performed single-cell sequencing on individual cells of ‘*Ca*. Omnitrophus magneticus’ SKK-01 (SKK-01), Mbav, and ‘*Ca*. Magnetoovum chiemensis’ CS-04 (CS-04) and obtained single amplified genomes (SAGs) for each populations. They then combined six, six, and four SAGs from SKK-01, Mbav, and CS-04 cells to obtain the draft genomes with a completeness of 74%, 75%, and 87% for these three strains, respectively. These data, together with this study, demonstrate the great importance and efficiency of the target-specific mini-metagenomics technique in obtaining draft genomes of uncultured environmental MTB, especially those with low abundance.

MGCs are the essential genes for magnetosome biosynthesis and their cellular localization. ANI analysis suggests that LHC-1 and XYR belong to the same species. Thus, the gene content and organization of MGCs between LHC-1 and XYR are highly conserved (Fig. 4), except that the MGC of LHC-1, but not of XYR, contains the *mad29* and *feoB* genes, which are commonly identified in other MGCs of Nitrospirota MTB. For the MGC predicted in the LHC-1 draft genome, the *mam* genes of *mamM*, *-Q*, *-B*, *-I*, *-E*, and *-O* are conserved in almost all analyzed MTB strains and have been proposed to be essential membrane proteins for magnetosome membrane formation and growth during the early stage in the model *alphaproteobacterial* MTB strains AMB-1 and MSR-1 [79–83], indicating that the early stage magnetosome production of LHC-1 may be similar to that of AMB-1 and MSR-1, which produce cuboctahedral-shaped magnetic crystals. Little is known about the *mad* genes, which are probably involved in the production of bullet-shaped magnetite crystals [84]. One mystery of Nitrospirota MTB is why and how they produce such large amounts of magnetosomes and multiple bundles of magnetosome chains per cell. Our analysis suggests that the Man proteins may use enriched coiled-coil domains to organize the intricate multiple bundles of magnetosome chains. The exact functions of these *man* genes remain to be verified by genetic studies.

A combination of fluorescence microscopy and electron microscopy have been previously performed on cultivated [85] and uncultivated [86] MTB to investigate their biomineralization, morphology, and phylogeny. The NanoSIMS technique has been increasingly applied to the metabolic analysis of various environmental microbes, including anaerobic phototrophic bacteria [4], N_2_-fixing bacteria [87], phytoplankton [88], and microbe-host interactions [89–91]. The FISH-NanoSIMS coupled techniques have been used to study the nitrogen metabolism of marine nitrite-oxidizing bacteria [92] and the carbon metabolism of an autotrophic, nitrate-reducing, Fe(II)-oxidizing enrichment culture [93]. For MTB analysis, NanoSIMS has been conducted to analyze the biomineralization of magnetosomes in cultured strains *Desulfovibrio magneticus* strain RS-1 [94] and MSR-1 [95]. Recently, a correlative approach of SIP (stable isotope probing)-FISH-Raman-SEM-NanoSIMS has been developed and further applied to characterize a population of uncultivated multicellular magnetotactic bacteria (MMB) producing Fe_3_S_4_ magnetosomes belonging to the phylum Desulfobacterota [96]. The authors used NanoSIMS to characterize D_2_O uptake and the magnetosome distribution (localization of Fe and S) within the multicellular MTB [96]. More recently, individual MMB consortia were separated and sequenced, followed by SIP-FISH-NanoSIMS to test the genomic predictions, which simultaneously provides information on MMB diversity, ecology, genomics, and physiology [97]. In our case, we combined target-specific mini-metagenomics and stable-isotope analysis by cooperating single-cell sorting and sequencing, FISH, FIB-SEM, stable-isotope incubation, and NanoSIMS techniques (Fig. 1), to link the identity, morphology, genome, and verified metabolisms of an uncultured Nitrospirota MTB LHC-1 at the single-cell level.

### LHC-1 and many other MTB from different phyla are autotrophs

Nitrospirota MTB are widespread and quite abundant in various aquatic ecosystems and have been proposed to play an important role in the biogeochemical cycles of C, N, S, P, Fe, etc. [8, 12–14, 16–19, 21]. Several Nitrospirota MTB from different genera have been reported to contain the genetic potential for CO_2_ fixation via the WL and/or rTCA pathways [16, 22, 23, 58]. In this study, we predicted and verified the ability of CO_2_ fixation in an uncultured Nitrospirota MTB LHC-1. Besides Nitrospirota MTB, the cultivated Pseudomonadota MTB strains *Magnetovibrio blakemorei* MV-1 (MV-1) [98], *Magnetospira thiophila* MMS-1 (MMS-1) [99], and *Magnetococcus marinus* MC-1 (MC-1) [100] are also capable of using CO_2_ as a sole carbon source. The MV-1 strain possesses a type II ribulose-1,5-bisphosphate carboxylase/oxygenase (RubisCO) gene (*cbbM*) and uses the Calvin-Benson-Bassham (CBB) cycle for CO_2_ fixation and autotrophy [98]. MMS-1 also uses the CBB cycle for CO_2_ fixation and autotrophy [99]. During autotrophic growth, MC-1 relies on the rTCA cycle for CO_2_ fixation [100]. Although LHC-1 has most of the genes involved in the CBB cycle, the predicted RubisCO is type IV, which could catalyze reactions other than RuBP carboxylation and may not be functional for carbon fixation [101], so LHC-1 may not use the CBB pathway for carbon fixation. Similarly, genes encoding type IV RubisCO were found in both the Mbav and Mcas genomes [13, 22, 23]. These data indicate that MTB from different phyla can use CO_2_ as a carbon source through different carbon fixation pathways, suggesting an important role of MTB in the inorganic carbon cycling.

The ability of nitrate reduction has also been proposed in several Nitrospirota MTB species by genomic prediction [16, 22, 58]. Based on the obtained draft genome of LHC-1, we predicted that LHC-1 could use NO_3_^−^ as a nitrogen source by the denitrification pathway and/or dissimilatory nitrate reduction to ammonium, which was tested by NanoSIMS analysis, indicating that LHC-1 could use nitrate as a terminal electron acceptor. For the cultured Alphaproteobacteria *Magnetospirillum magnetotacticum* MS-1 (MS-1), when grown under conditions where NO_3_^−^ is the sole nitrogen source, they simultaneously carry out denitrification to N_2_ and dissimilatory nitrate reduction to ammonium [102]. Interestingly, magnetite biomineralization and anaerobic growth have been experimentally demonstrated to be closely related to the denitrification process in the cultured model Alphaproteobacteria strains MSR-1 and AMB-1 [103, 104]. These data suggest that the ability to use NO_3_^−^ as a nitrogen source may be conserved in many MTB groups and plays an important role in magnetosome biomineralization.

### The localization of C, N, O, and S is specific in LHC-1

Both the C and N signals are mainly distributed around the cell periphery of LHC-1 (Fig. 7). Much of the denitrification process of gram-negative bacteria has been found to be restricted to the periplasm [73]. The reduction of NO_3_^−^ to NO_2_^−^ is the first step in the utilization of NO_3_^−^ and is thought to be catalyzed mainly by the periplasmic NO_3_^−^ reductase complex NapAB and partly by a membrane-bound NO_3_^−^ reductase NarGHI [105]. Then, a periplasmic NO_2_^−^ reductase NirS or NirK catalyzes the reduction of NO_2_^−^ to NO, which is further reduced to N_2_O by a NO reductase NorBC that is an integral membrane protein with its active site in the periplasm. Finally, a periplasmic N_2_O reductase (Nos) catalyzes the reduction of N_2_O to N_2_ [105]. The genome of LHC-1 contains genes encoding NapAB, NarGHI, NirS, NorBC, and NosZ (Fig. 5), indicating that the denitrification process of LHC-1 most likely occurred in the periplasm, and the dissimilatory nitrate reduction pathway might occur close to the bacterial inner membrane, which was demonstrated in our study with the predominant location of nitrogen isotopes around the cell (Fig. 7). The WL pathway and the rTCA cycle are most likely to occur in the cytoplasm of the bacteria; thus far, the reason for the bacteria peripheral localization of the newly fixed carbon in LHC-1 is still unclear. One possible reason is that some stable-isotope-labeled carbon may passively diffuse through the bacteria outer and inner membrane into the cytoplasm.

The colocalization of the major O signal with the magnetosome chain in the SEM images (Fig. 7) confirms that Nitrospirota MTB LHC-1 produces magnetite (Fe_3_O_4_) magnetosomes. However, it is not clear why the intensity and distribution of the S signal are very similar to the ^12^C^14^N^−^ signal at different incubation times. Since no obvious sulfur granules were seen in these cells on the SEM images, the ^32^S^−^ signals may represent the distribution of sulfur-containing proteins and other sulfur-rich compounds in the LHC-1 cells.

### The heterogeneity and dynamics of C, N, and S metabolism in the LHC-1 cell population

In this study, the LHC-1 strain displayed a physiological heterogeneity from cell to cell, including cell morphology and metabolism. As mentioned above, we observed morphological heterogeneity of sulfur granules in LHC-1 cells from SEM images (Fig. S2). Several uncultured Nitrospirota MTB species have been reported to accumulate sulfur granules in the cytoplasm and participate in the microbial sulfur cycling across the aquatic oxic-anoxic interface based on magnetotaxis [13, 106–108]. The morphological heterogeneity of sulfur inclusions has been discovered in the Nitrospirota MTB strain Mbav [13] by electron microscopy observations. In other words, in the same Nitrospirota MTB population, the cells might contain different numbers of sulfur globules in the cytoplasm, probably due to the different metabolic status that the intracellular sulfur inclusions serve as a reservoir for further oxidation as previously proposed [10, 13, 22].

Moreover, the heterogeneity of C and N uptake in LHC-1 was also observed. The dots (each dot represents a single cell) on the scatter plot about ^13^C^−^/^12^C^−^ versus ^15^N^−^/^14^N^−^ (Fig. 8c) were very concentrated and near to the natural values at 0 hpi. With the addition and incubation of H^13^CO_3_^−^ and ^15^NO_3_^−^, the dots on the scatter plot became more and more dispersed, indicating that the heterogeneity of C and N uptake in LHC-1 increased with incubation time. Interestingly, the dots spread more on the *y*-axis (^15^N^−^/^14^N^−^) than on the *x*-axis (^13^C^−^/^12^C^−^), especially at 17.5 hpi, which may represent a unique metabolic state or growth stage. These data indicate that the cell-to-cell heterogeneity of LHC-1 in N uptake is likely greater than in C uptake.

The overall ratios of C and N uptake showed an increase in the trend at the beginning of the incubation period and a plateau phase towards the late incubation period. One striking time point is the ^15^N^−^/^14^N^−^ ratio at 17.5 hpi, which might due to the fact that the potential of the dissimilatory nitrate reduction pathway (NO^3−^→NO^2−^→NH^4+^) is higher than that of the denitrification pathway (NO_3_^−^→NO_2_^−^→NO→N_2_O→N_2_). The decrease in the ^15^N^−^/^14^N^−^ ratio from 17.5 to 23 hpi could be the opposite of what happened between 16.5 and 17.5 hpi. The decrease and increase in ^13^C^−^/^12^C^−^ and ^15^N^−^/^14^N^−^ uptake occurred at later time points, when the LHC-1 population has higher cell-to-cell heterogeneity and limited time point data, so the correlations between C and N metabolism and the different growth stages of LHC-1 remain to be explored.

## Conclusion

Linking microbial genomic potential to actual metabolism in the environment is challenging, especially at the single-cell level. In this study, we developed a workflow to simultaneously investigate the identity, morphology, genome, and metabolism of environmental MTB at the single-cell level. Our results show that the uncultured Nitrospirota MTB strain ‘*Ca.* Magnetobacterium’ sp. LHC-1 can convert inorganic carbon and nitrogen into biomass and energy through CO_2_ fixation and NO_3_^−^ reduction. This indicates that LHC-1 is an autotroph and makes a contribution to the cycling of C and N in addition to Fe in the natural environment. We observed the temporal dynamics of C and N uptake in the LHC-1 population, which correlated with their growth status. Cultivation of Nitrospirota MTB cells would further help to quantify their contributions to C and N cycling. Together, the combination of different techniques (i.e., target-specific mini-metagenomics, FISH, FIB-SEM, and NanoSIMS) is a promising strategy to comprehensively study the mechanisms of microbe-environment interactions.

### Supplementary Information

Additional file 1: Supplementary figures and tables. Fig. S1 The cell sorting process with micromanipulation system in step 1 of Fig. 1. About 100 μl of filtered sample water (using 0.22 μm membrane filter) was added beside the sediment on a glass slide for easy single-cell extraction. Under the magnetic field created by magnets beside the glass slide, north-seeking MTB could swim to the left edge of the water droplet from the sediment. Then the individual potential LHC-1 cells were selected and picked up using a single capillary needle and washed four times in the four drops of liquid on the glass slide. The cells were washed two times with filtered sample water, and two times with sterilized PBS buffer. Finally, different numbered cell groups were collected for single-cell sequencing. Fig. S2 SEM image of representative LHC-1 cells that are without (a), with few (b), and full of (c) sulfur granules. The magnetosome chains (pointed by yellow arrows) are presented in white color. The potential sulfur granules (some are pointed by yellow arrowheads) are presented as white globules. Fig. S3 Scatter plot of cell length versus cell width of LHC-1 at eight incubation time points. Each dot represents a single cell. The red dashed lines and numbers at each time point show the average cell length and width values. Table S1 Topology and domain prediction of Man1 to Man6 proteins. Table S2 Cell size and the isotope ratio range of the LHC-1 cells at different incubation time points.

Additional file 2: Supplementary file data sheet 1. 120 bacterial single-copy concatenated protein sequence alignments.

Additional file 3: Supplementary file data sheet 2. ANI analysis of LHC-1 and available Nitrospirota MTB genomes.

Additional file 4: Supplementary file data sheet 3. 10 magnetosome‐associated membrane (*mam*) genes.

## Data Availability

The genome sequence of LHC-1 has been deposited in GenBank under the BioProject number PRJNA400260 (BioSample accession number SAMN38167836) and the National Microbiology Data Center (NMDC) with accession number NMDC60147459 under the project NMDC10017683 (http://nmdc.cn).

## Abbreviations

MTB: Magnetotactic bacteria
NanoSIMS: Nanoscale secondary ion mass spectrometry
AMB-1: *Magnetospirillum magneticum* strain AMB-1
MSR-1: *Magnetospirillum gryphiswaldense* strain MSR-1
TEM: Transmission electron microscopy
OTU: Operational taxonomic units
MDA: Multiple displacement amplification
FISH: Fluorescence *in situ* hybridization
*E. coli*: *Escherichia coli*
SEM: Scanning electron microscope
FIB: Focused ion beam
Pt: Platinum
ROI: Regions of interest
LHC-1: Lianhuachi-1
LHC-2: Lianhuachi-2
XYR: ‘*Candidatus* Magnetobacterium cryptolimnobacter’
Mcas: ‘*Candidatus* Magnetobacterium casensis’
Mbav: ‘*Candidatus* Magnetobacterium bavaricum’
ANI: Average nucleotide identity
MGC: Magnetosome gene cluster
*mam*: Magnetosome-associated membrane genes
*mad*: Magnetosome-associated Deltaproteobacteria genes
*man*: Magnetosome‐associated Nitrospirota genes
WL: Wood-Ljungdahl pathway
rTCA: Reductive tricarboxylic acid cycle
TCA: Tricarboxylic acid cycle
hpi: Hour post-incubation
SKK-01: ‘*Candidatus* Omnitrophus magneticus’ SKK-01
CS-04: ‘*Candidatus* Magnetoovum chiemensis’ CS-04
SAGs: Single amplified genomes
MV-1: *Magnetovibrio blakemorei* MV-1
MMS-1: *Magnetospira thiophila* MMS-1
MC-1: *Magnetococcus marinus* MC-1
RubisCO: Ribulose-1, 5-bisphosphate carboxylase/oxygenase
