# Supplementary material for: Linking morphology, genome, and metabolic activity of uncultured magnetotactic Nitrospirota at the single-cell level
Source: Microbiome. 2024 Aug 24;12:158. doi: 10.1186/s40168-024-01837-6 (PMC11344931; doi:10.1186/s40168-024-01837-6)
Supplement: Supplementary file 2 — Additional file 1: Supplementary figures and tables. Fig. S1 The cell sorting process with micromanipulation system in step 1 of Fig. 1. About 100 μl of filtered sample water (using 0.22 μm membrane filter) was added beside the sediment on a glass slide for easy single-cell extraction. Under the magnetic field created by magnets beside the glass slide, north-seeking MTB could swim to the left edge of the water droplet from the sediment. Then the individual potential LHC-1 cells were selected and picked up using a single capillary needle and washed four times in the four drops of liquid on the glass slide. The cells were washed two times with filtered sample water, and two times with sterilized PBS buffer. Finally, different numbered cell groups were collected for single-cell sequencing. Fig. S2 SEM image of representative LHC-1 cells that are without (a), with few (b), and full of (c) sulfur granules. The magnetosome chains (pointed by yellow arrows) are presented in white color. The potential sulfur granules (some are pointed by yellow arrowheads) are presented as white globules. Fig. S3 Scatter plot of cell length versus cell width of LHC-1 at eight incubation time points. Each dot represents a single cell. The red dashed lines and numbers at each time point show the average cell length and width values. Table S1 Topology and domain prediction of Man1 to Man6 proteins. Table S2 Cell size and the isotope ratio range of the LHC-1 cells at different incubation time points. [file 40168_2024_1837_MOESM1_ESM.docx]

**SUPPLEMENTARY INFORMATION**

**Linking morphology, genome and metabolic activity of uncultured magnetotactic *Nitrospirae* at the single-cell level**

Runjia Ji^1,2,3#^, Juan Wan^1,2#^, Jia Liu^2,4^, Jinbo Zheng^1,3,5^, Tian Xiao ^2,4^, Yongxin Pan^1,2,3^, and Wei Lin^1,2,3*^

1 Key Laboratory of Earth and Planetary Physics, Institute of Geology and Geophysics, Chinese Academy of Sciences, 100029, Beijing, China.

2 France-China Joint Laboratory for Evolution and Development of Magnetotactic Multicellular Organisms, Chinese Academy of Sciences, 100029, Beijing, China.

3 College of Earth and Planetary Sciences, University of Chinese Academy of Sciences, 100049, Beijing, China.

4 Key Laboratory of Marine Ecology and Environmental Sciences, Institute of Oceanology, Chinese Academy of Sciences, Qingdao, China

5 Engineering Laboratory for Deep Resources Equipment and Technology, Institute of Geology and Geophysics, Chinese Academy of Sciences, Beijing 100029, China.

^#^These authors contributed equally to this work

^*^Corresponding author: Wei Lin

Email: [weilin@mail.iggcas.ac.cn](mailto:weilin@mail.iggcas.ac.cn)

**
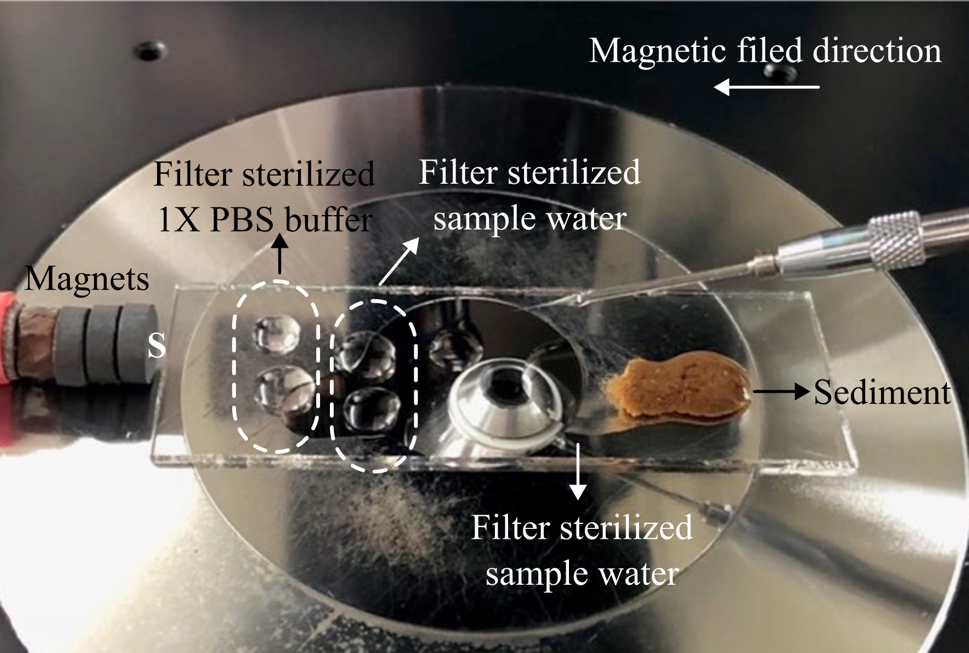
**

**Fig. S1 The cell sorting process with micromanipulation system in step 1 of Fig. 1**. About 100 μl of filtered sample water (using 0.22 μm membrane filter) was added beside the sediment on a glass slide for easy single-cell extraction. Under the magnetic field created by magnets beside the glass slide, north-seeking MTB could swim to the left edge of the water droplet from the sediment. Then the individual potential LHC-1 cells were selected and picked up using a single capillary needle and washed four times in the four drops of liquid on the glass slide. The cells were washed two times with filtered sample water, and two times with sterilized PBS buffer. Finally, different numbered cell groups were collected for single-cell sequencing.

**Fig. S2 SEM image of representative LHC-1 cells that are without (a), with few (b), and full of (c) sulfur granules.** The magnetosome chains (pointed by yellow arrows) are presented in white color. The potential sulfur granules (some are pointed by yellow arrowheads) are presented as white globules.


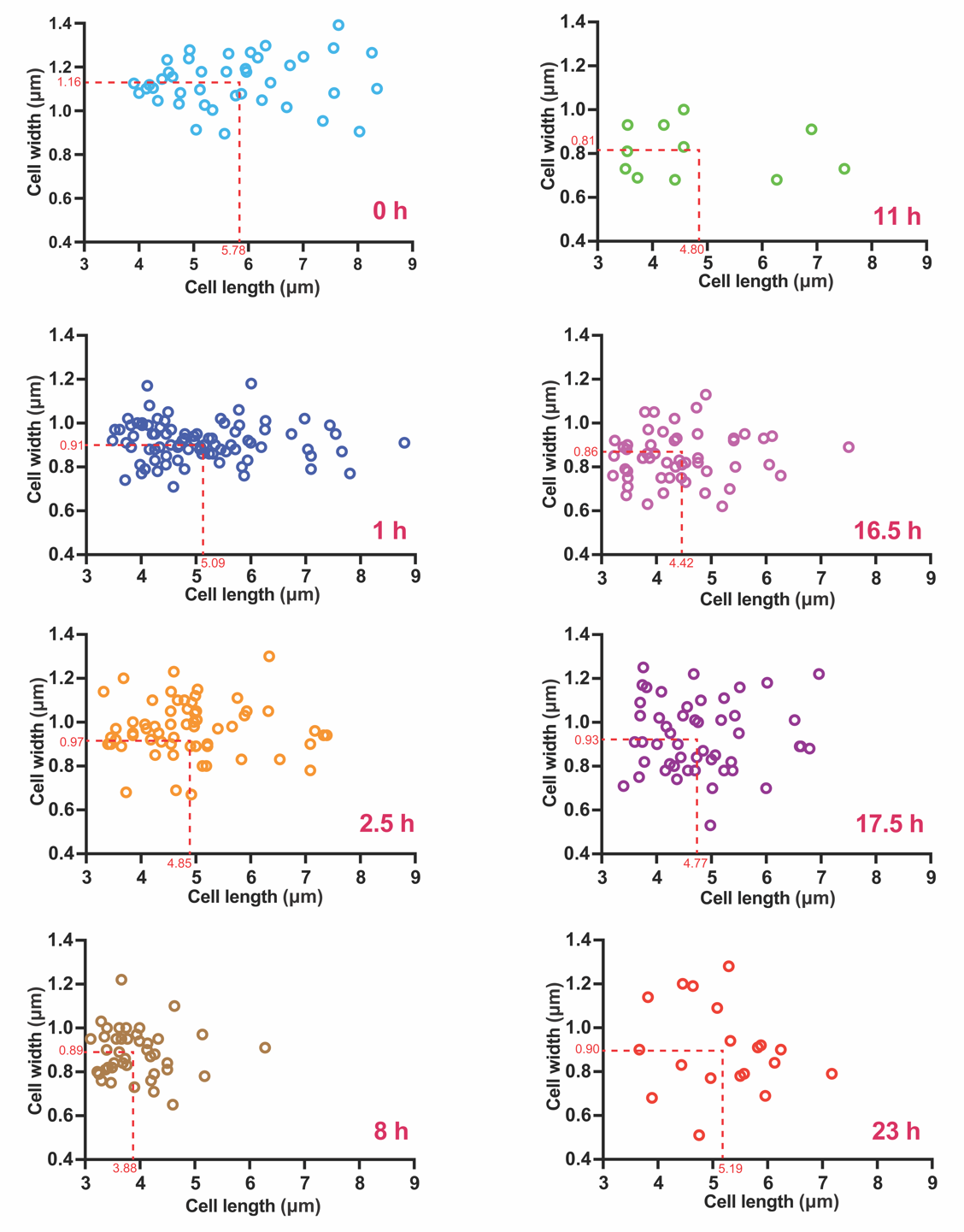


**Fig. S3** **Scatter plot of cell length versus cell width of LHC-1 at eight incubation time points.** Each dot represents a single cell. The red dashed lines and numbers at each time point show the average cell length and width values.

**Table S1 Topology and domain prediction of Man1 to Man6 proteins**

| **Gene name** | **CCTOP** | **SMART** | **InterProScan** |
| --- | --- | --- | --- |
| **Man1** | No TM | Coiled-coil domain at 123-151 | Coiled-coil domain at 131-151 |
| **Man2** | No TM | TM domain at 5-27 and 42-64 | 1. Lipopolysaccharide assembly protein A (LapA) domain at 21-72;  2. TM domain at 5-27 and 42-64 |
| **Man3** | No TM | TM domain at 4-23 | 1. Signal peptide at 1-18;  2. No cytoplasmic domain at 19-50;  3. TM domain at 4-23 |
| **Man4** | No TM | Coiled-coil domain at 54-140 | Coiled-coil domain at 58-99 and 111-138 |
| **Man5** | No TM | Coiled-coil domain at 12-49, 87-121, 157-224, and 278-329 | Coiled-coil domain at 17-48, 93-120, 162-196, 204-224, and 301-325 |
| **Man6** | No TM | Coiled-coil domain at 65-96, 185-306 | Coiled-coil domain at 70-90, 111-131, 146-180, 187-249, and 266-300 |

CCTOP, Constrained Consensus Topology prediction server (integrates the prediction from several prediction methods: HMMTOP, Membrain, Memsat-SVM, Octopus, Philius, Phobius, Pro, Prodiv, Scampi, and TMHMM). TM, transmembrane domain.

**Table S2 Cell size and the isotope ratio range of the LHC-1 cells at different incubation time points.**

| **Incubation time (h)** | **Number of cells** | **Cell length (μm)** | **Cell width (μm)** | **Cell volume (μm^3^)** | **Isotope ratio range %** | |
| --- | --- | --- | --- | --- | --- | --- |
|  |  |  |  |  | **^13^C^-^/^12^C^-^** | **^15^N^-^/^14^N^-^** |
| **0** | 4 | 3.91 - 8.26 (5.67 ± 1.86) | 1.12 - 1.28 (1.21 ± 0.07) | 3.51 - 9.85 (6.21 ± 2.65) | 0.86 - 0.89 (0.88 ± 0.02) | 0.22 - 0.29 (0.26 ± 0.03) |
| **1** | 86 | 3.48 - 8.8 (5.09 ± 1.13) | 0.71 - 1.18 (0.91 ± 0.09) | 1.49 - 6.14 (3.16 ± 0.92) | 0.93 - 1.08 (1.0 ± 0.03) | 0.35 - 0.44 (0.38 ± 0.01) |
| **2.5** | 62 | 2.99 - 7.39 (4.85 ± 1.05) | 0.67 - 1.3 (0.97 ± 0.13) | 1.27 - 7.84 (3.39 ± 1.14) | 0.99 - 1.35 (1.1 ± 0.08) | 0.36 - 0.53 (0.4 ± 0.02) |
| **8** | 44 | 2.97 - 6.28 (3.88 ± 0.64) | 0.65 - 1.22 (0.89 ± 0.11) | 1.38 - 4.05 (2.24 ± 0.66) | 1.02 - 1.25 (1.12 ± 0.05) | 0.41 - 1.17 (0.6 ± 0.19) |
| **11** | 11 | 3.51 - 7.5 (4.8 ± 1.43) | 0.68 - 1.0 (0.81 ± 0.12) | 1.31 - 4.29 (2.35 ± 0.92) | 1.02 - 1.62 (1.24 ± 0.17) | 0.38 - 0.71 (0.49 ± 0.1) |
| **16.5** | 54 | 3.21 - 7.51 (4.42 ± 0.9) | 0.62 - 1.59 (0.86 ± 0.15) | 1.13 - 7.76 (2.47 ± 1.12) | 0.68 - 1.42 (1.1 ± 0.14) | 0.39 - 1.03 (0.52 ± 0.13) |
| **17.5** | 50 | 3.4 - 6.96 (4.77 ± 0.91) | 0.53 - 1.25 (0.93 ± 0.16) | 1.06 - 7.66 (3.14 ± 1.28) | 0.86 - 1.48 (1.16 ± 0.14) | 0.38 - 2.11 (0.99 ± 0.46) |
| **23** | 19 | 3.66 - 7.17 (5.19 ± 0.92) | 0.51 - 1.28 (0.9 ± 0.2) | 0.94 - 6.26 (3.2 ± 1.28) | 1.04 - 1.5 (1.21 ± 0.13) | 0.4 - 1.18 (0.56 ± 0.23) |
| **Total** | 330 | 2.97 - 8.8 (4.73 ± 1.07) | 0.51 - 1.59 (0.91 ± 0.14) | 0.94 - 9.85 (2.98 ± 1.2) | 0.68 - 1.62 (1.09 ± 0.12) | 0.22 - 2.11 (0.54 ± 0.29) |

Note: the values from columns 3-7 show the minimum to the maximum range with the average in the parentheses (mean ± SD). The cell volume was calculated assuming that the shape of LHC-1 cell is a cylinder with one hemisphere at each end.
